# Noninferiority Study Comparing Latanoprost 0.005% Without Versus With Benzalkonium Chloride in Open-Angle Glaucoma or Ocular Hypertension

**DOI:** 10.1097/ICL.0000000000000860

**Published:** 2021-11-17

**Authors:** David Wirta, Ranjan Malhotra, James Peace, Bridgitte Shen Lee, Brittany Mitchell, Kenneth Sall, Matthew McMenemy

**Affiliations:** Eye Research Foundation (D.W.), Newport Beach, CA; Ophthalmology Associates (R.M.), St. Louis, MO; Peace Eyecare (J.P.), Inglewood, CA; Vision Optique (B.S.L.), Houston, TX; Sun Pharmaceutical Industries, Inc., (B.M.) Princeton, NJ; Sall Research Medical Center, Inc., (K.S.) Artesia, CA; and Lone Star Eye Care, P.A (M.M.), Sugar Land, TX.

**Keywords:** Intraocular pressure, Glaucoma, Latanoprost, Benzalkonium chloride

## Abstract

**Objectives:**

To evaluate the noninferiority of intraocular pressure (IOP)-lowering latanoprost without benzalkonium chloride (BAK) versus latanoprost with BAK (for treatment of open-angle glaucoma or ocular hypertension).

**Methods:**

Overall, 578 patients were randomized 1:1 to latanoprost without BAK or latanoprost with BAK once daily in the affected eye(s) for 12 weeks. The primary efficacy endpoint was IOP, measured on days 0, 7, 28, 56, and 84 (8 am, 10 am, and 4 pm). Noninferiority was established if the following criteria were met: 95% confidence interval (CI) of the mean difference between treatments included 0 mm Hg for all time points (N1), 95% CI upper limit less than 1.5 mm Hg (N2), and less than 1 mm Hg for≥7 of 12 time points (N3). Primary efficacy analysis was performed on the intent-to-treat population. Safety measurements included ocular and systemic adverse event (AE).

**Results:**

The 95% CI included 0 mm Hg for 7/12 time points (N1), 95% CI upper limit was less than 1.5 mm Hg for 12/12 time points (N2), and less than 1.0 mm Hg for 4/7 time points (N3). AEs were mild and similarly distributed between groups.

**Conclusions:**

Latanoprost without BAK did not meet two of three criteria for noninferiority and showed a similar safety profile relative to latanoprost with BAK.

## Abstract 2

Supplemental Digital Content is Available in the Text.

Glaucoma is a chronic, incurable ophthalmic disorder and one of the leading global causes of vision impairment and blindness. In 2015, an estimated four million adults worldwide suffered glaucoma-related vision impairment and another 2.9 million from glaucoma-related blindness.^1^ Risk factors for open-angle glaucoma (OAG) include increased intraocular pressure (IOP); reduction of IOP is an essential part of glaucoma management and can slow disease progression.^2^

Prostanoid selective FP receptor agonists increase the outflow of aqueous humor, which decreases IOP.^3^ Latanoprost 0.005% ophthalmic solution (Xalatan; Pharmacia & Upjohn Co, New York, NY) includes 0.02% benzalkonium chloride (BAK) as a preservative and is approved for the reduction of IOP in patients with OAG or ocular hypertension (OHT).^4^ BAK may increase epithelial cell apoptosis and eye irritation, decrease epithelial cell integrity, and increase the risk of allergies and delayed hypersensitivity reactions.^5^ Long-term exposure to BAK may cause conjunctival scarring.^5^ The use of contact lenses is contraindicated if the patient is on medication containing BAK, because BAK may accumulate in the lenses over time.^4^ Several alternatives without BAK are available, including travoprost 0.004%. A phase 3 study in patients with OAG or OHT compared travoprost with BAK to travoprost without BAK; the formulation without BAK was equally effective as the formulation with BAK, and no clinically relevant difference was found regarding adverse events (AEs).^6^

Despite its downside, BAK may display great ocular penetration capacity,^7^ making it the preservative of choice in topical ophthalmic preparations, and creating a need for a more effective IOP-lowering medication without BAK. Latanoprost 0.005% ophthalmic emulsion without BAK (Xelpros; Sun Pharmaceutical Industries, Inc., Cranbury, NJ) is approved by the US Food and Drug Administration to reduce elevated IOP in patients with OAG or OHT.^8^ In this formulation, potassium sorbate is used as an effective and safe preservative alternative to BAK; in addition, Solutol HS 15, which demonstrates a lower histamine release profile in animal toxicity studies than a macroemulsion, is used as surfactant.^8,9^

Here, we present the results of a clinical study in patients with OAG or OHT (NCT00947661) evaluating the efficacy, safety, and noninferiority of latanoprost 0.005% without BAK (herein referred to as latanoprost without BAK) versus latanoprost 0.005% with BAK (Xalatan, herein referred to as latanoprost with BAK).

## METHODS

### Study Design

This was a randomized, assessor-masked, actively controlled, parallel-group study, which adhered to the Declaration of Helsinki and the International Council for Harmonisation Consolidated Guideline E6 for Good Clinical Practice. All patients provided written informed consent before enrollment.

Patients≥18 years with OHT or primary OAG could enroll if unmedicated IOP was≥22 mm Hg with≤5 mm Hg intereye difference at eligibility visit. Presence of pseudoexfoliation or pigment dispersion was acceptable. Additional inclusion criteria comprised Early Treatment Diabetic Retinopathy Study (ETDRS) visual acuity of 1.00 or better and a visual field defect defined as Humphrey Swedish Interactive Threshold Algorithm mean deviation greater than −20 dB and no central point depression to 0 dB. Patients had to be willing to refrain from using contact lenses for the duration of the study.

Patients were excluded if they had a history of allergic hypersensitivity or poor tolerance to any study compounds or a known lack of ocular hypotensive response to topical ophthalmic prostaglandin analogs. Patients were also excluded if they had undergone intraocular conventional or laser surgery within 6 months of the study or refractive surgery in the study eye within the past 3 months; had a diagnosis of angle closure glaucoma, progressive retinal or optic nerve disease apart from glaucoma, a ruptured posterior lens capsule, concurrent infectious or noninfectious conjunctivitis, keratitis, or uveitis; or have had a risk for macular edema. Other key exclusion criteria comprised central corneal thickness of greater than 620 μm in the study eye or any abnormality preventing stable applanation tonometry, and use of ocular medications other than ocular hypotensive drugs or lubricating drops within 30 days of baseline.

Concomitant medications known to alter IOP—including beta antagonists, alpha and beta agonists, miotics, carbonic anhydrase inhibitors, and ocular hypotensives—were not permitted during the study. Patients taking these medications completed a washout period before randomization, with a minimum duration of 4 weeks for beta-antagonists, 1 week for topical corticosteroids, and 72 hrs for all other IOP-altering medications.

After the washout period, patients were randomized 1:1 to receive latanoprost without BAK or latanoprost with BAK. Randomization was stratified using a block randomization scheme with a block size of four patients and a stratification factor of baseline IOP defined as low IOP [22–28 mm Hg] and high IOP [29–35 mm Hg] in the study eye. The study eye was defined as the eye with higher IOP at the randomization visit. If IOP in both eyes was equal, the left eye was selected in patients with an even randomization number and the right eye was selected in patient with an odd number.

**FIG. 1.**
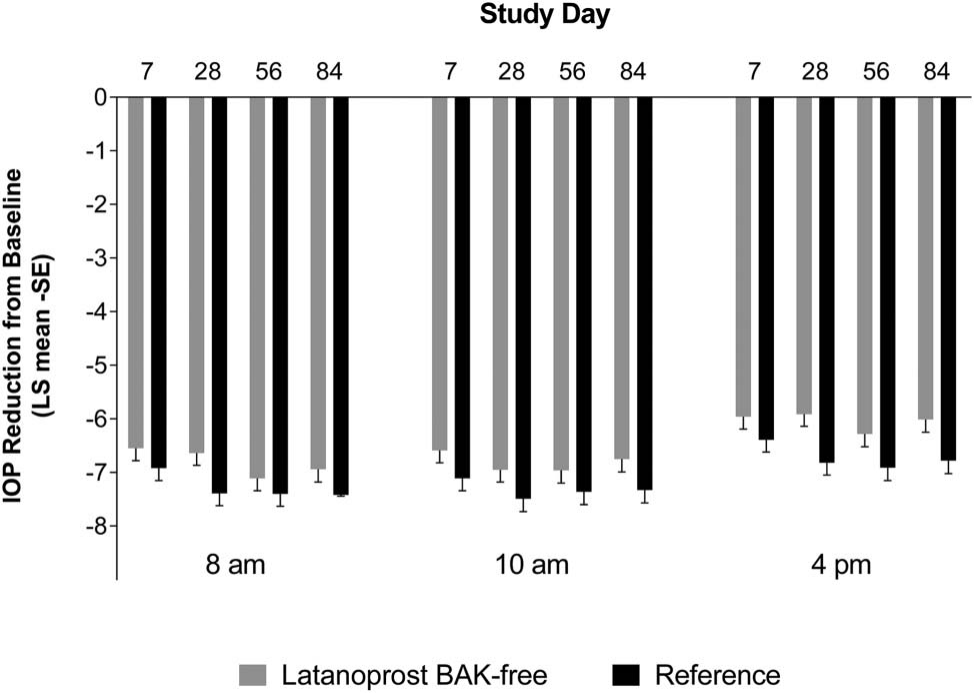
LS mean intraocular pressure reduction from baseline (-SE). benzalkonium chloride (BAK); intraocular pressure (IOP); least squares (LS); standard error (SE).

A total of seven study visits were conducted over 12 weeks: screening (day−35), eligibility and randomization (day−7), baseline (day 0), visit 1 (day 7±1 day), visit 2 (day 28±1 day), visit 3 (day 56±1 day), and visit 4 (end-of-study visit, day 84±1 day) (see Figure 1, Supplemental Digital Content 1, http://links.lww.com/ICL/A193). Patients received 1 drop of latanoprost without BAK or latanoprost with BAK once daily at approximately 8 pm.

### Efficacy Assessments

The primary efficacy endpoint was the change from baseline in IOP, which was measured at 8 am, 10 am, and 4 pm at baseline and at study visits 1 to 4. Each IOP measurement was performed in triplicate using a Goldmann applanation tonometer, and the average of the triplicate was taken as the IOP measurement for that time point. Change from baseline in IOP for each postbaseline time point was calculated using the IOP measurement for the corresponding time of day during the baseline visit.

### Safety Assessments

The primary safety assessment was ocular and systemic AE monitoring and reporting. Safety assessments also included resting pulse rate and blood pressure, ETDRS visual acuity, and slitlamp biomicroscopy, which were performed at each visit. Conjunctival hyperemia was assessed at the eligibility and randomization visit, baseline, and study visits 1 to 4, using a 0 to 3 scale (0=none, 1=mild, 2=moderate, 3=severe) using the 4-point Ora Calibra Dry Eye Redness Scale. Dilated ophthalmoscopy and Humphrey visual field assessments were performed at screening and end-of-study visits.

### Statistical Analyses

To establish noninferiority, the following three criteria had to be met simultaneously for treatment differences in IOP: the 95% confidence interval (CI) of the least squares (LS) mean difference between treatments included 0 mm Hg at all 12 time points (N1); the upper limit of the 95% CI was less than 1.5 mm Hg at all 12 time points (N2); and the upper limit of the 95% CI was less than 1.0 mm Hg for≥7 of the 12 time points (N3).

The intent-to-treat (ITT) population included all randomized patients. The safety population included all patients who enrolled and received at least 1 dose of study medication. The per-protocol population included all patients who had completed the end-of-study visit and did not have any major protocol violations.

Sample size was determined based on a test of noninferiority and assumed a dropout rate of 10% and a SD of 3.5 mm Hg in IOP reduction from baseline. A total of 578 patients were enrolled and randomized in a 1:1 ratio to have at least 259 evaluable patients per treatment arm. The primary efficacy analysis was performed on observed data for the ITT population without imputation of missing data. An additional analysis was performed on the per-protocol population. The impact of missing data on the efficacy conclusion was assessed using three different data imputation methods, namely last observation carried forward (LOCF), baseline observation carried forward, and multiple imputations. Efficacy analysis was also performed on the subgroups of patients with low (22–28 mm Hg) and high (29–35 mm Hg) baseline IOP. Mean change from baseline IOP was calculated as LS mean using an analysis of covariance model that includes treatment, site, and baseline IOP group as a covariate. A two-sided 95% CI for the difference between latanoprost without BAK and latanoprost with BAK was derived at each time point.

Baseline demographic data were reported for the ITT population using summary statistics. Safety data were reported for the safety population using summary statistics.

## RESULTS

### Patient Disposition and Baseline Characteristics

A total of 289 patients were randomized to each treatment group (latanoprost without BAK or latanoprost with BAK). Of these, 550 (95.2%) patients completed the study (see Figure 2, Supplemental Digital Content 1, http://links.lww.com/ICL/A194). Demographic characteristics were well balanced between the two treatment groups and are summarized in Table 1. Overall, most patients in the study were female (63.4%) and White (63.4%), with a mean±SD age of 63.4±10.4 years.

**TABLE 1.** Patient Demographics and Baseline Characteristics

Parameter	Latanoprost Without BAK (n=289)	Latanoprost With BAK (n=289)	Total (N=578)
Age, mean±SD (years)	63.8±11.1	63.1±9.6	63.4±10.4
Sex			
Female	188 (65.1)	186 (64.4)	374 (64.7)
Male	101 (34.9)	103 (35.6)	204 (35.3)
Race			
White	198 (68.5)	202 (69.9)	400 (69.2)
Black or African American	82 (28.4)	79 (27.3)	161 (27.9)
American Indian or Alaskan native	1 (0.3)	1 (0.3)	2 (0.3)
Asian	7 (2.4)	6 (2.1)	13 (2.2)
Other	1 (0.3)	1 (0.3)	2 (0.4)
Ethnicity			
Hispanic or Latino	53 (18.3)	54 (18.7)	107 (18.5)
Not Hispanic or Latino	236 (81.7)	235 (81.3)	471 (81.5)
Baseline IOP			
Low IOP group (22–28 mm Hg)	235 (81.3)	235 (81.3)	470 (81.3)
High IOP group (29–35 mm Hg)	54 (18.7)	54 (18.7)	108 (18.7)
IOP, mean±SD (mm Hg)			
Low IOP group (22–28 mm Hg)	23.8±1.6	23.9±1.6	23.9±1.6
High IOP group (29–35 mm Hg)	31.0±2.2	30.7±2.0	30.9±2.1
ETDRS visual acuity, mean±SD	0.1±0.1	0.1±0.1	0.1±0.1
Visual field mean deviation, mean±SD	−2.4±3.6	−1.8±3.1	−2.1±3.4
Cup-to-disk ratio, mean±SD	0.5±0.2	0.5±0.2	0.5±0.2
Diagnosis, n (%)^a^			
Ocular hypertension	104 (36.0)	97 (33.6)	201 (34.8)
Pseudoexfoliation	1 (0.3)	2 (0.7)	3 (0.5)
Pigment dispersion	3 (1.0)	5 (1.7)	8 (1.4)
Primary open angle glaucoma	193 (66.8)	200 (69.2)	393 (68.0)

Data shown for the ITT population. Data shown as n (%) unless otherwise noted. IOP measurements are for the study eye.

a Value may add up to more than 100% as a patient could have more than 1 diagnosis.

BAK, benzalkonium chloride; ETDRS, early treatment diabetic retinopathy study; IOP, intraocular pressure; ITT, intent-to-treat.

There were no important differences in baseline characteristics of the study eye between treatment groups. The mean±SD IOP was 23.8±1.6 vs 23.9±1.6 mm Hg in the low IOP group and 31.0±2.2 vs 30.7±2.0 mm Hg in the high IOP group for the latanoprost without BAK and latanoprost with BAK groups, respectively (Table 1).

### Primary Efficacy Analysis

Patients receiving latanoprost without BAK and latanoprost with BAK both achieved substantial and clinically meaningful reductions in IOP from baseline (Fig. 1). The reduction from baseline at all timepoints for latanoprost without BAK was approximately 6 to 7 mm Hg; the mean (95% CI) difference in IOP reduction from baseline between latanoprost with BAK and latanoprost without BAK ranged from 0.29 (−0.27, 0.85) to 0.91 (0.36, 1.47) mm Hg (Table 2). The 95% CI of the mean difference in IOP reduction from baseline included 0 mm Hg for seven of 12 required time points (N1). The upper limit of the 95% CI was less than 1.5 mm Hg for 12 of 12 required time points (N2). The upper limit of the 95% CI was less than 1.0 mm Hg for four of seven required time points (N3). Based on the predefined criteria, the noninferiority of latanoprost BAK-free was not established.

**TABLE 2.** Noninferiority Analysis Comparing Latanoprost Without BAK vs. Latanoprost With BAK

Time	Study Day	IOP Difference Between Treatment Groups (95% CI)	Noninferiority Criteria Met		
			N1	N2	N3
8 am	7	0.37 (−0.18, 0.92)	Y	Y	Y
	28	0.75 (0.19, 1.31)	N	Y	N
	56	0.29 (−0.27, 0.85)	Y	Y	Y
	84	0.48 (−0.10, 1.06)	Y	Y	N
10 am	7	0.52 (−0.03, 1.07)	Y	Y	N
	28	0.54 (−0.03, 1.10)	Y	Y	N
	56	0.40 (−0.17, 0.97)	Y	Y	Y
	84	0.58 (0.00, 1.17)	N	Y	N
4 pm	7	0.43 (−0.12, 0.97)	Y	Y	Y
	28	0.91 (0.36, 1.47)	N	Y	N
	56	0.63 (0.05, 1.20)	N	Y	N
	84	0.77 (0.20, 1.34)	N	Y	N
No. of times noninferiority criterion met			7	12	4
Minimum time points noninferiority criteria must be met			12	12	7

Data based on the ITT population.

For latanoprost without BAK to be considered noninferior to latanoprost with BAK, all three noninferiority criteria N1−N3 must be met for the minimum required time points.

N1, 95% CI includes 0 mm Hg; N2, the upper limit of the 95% CI is less than 1.5 mm Hg; N3, the upper limit of the 95% CI is less than 1 mm Hg.

BAK, benzalkonium chloride; CI, confidence interval; IOP, intraocular pressure; ITT, intent-to-treat.

The analysis of the per-protocol population was consistent with that of the ITT population; noninferiority was not established for the per-protocol population without LOCF (see Table 1, Supplemental Digital Content 1, http://links.lww.com/ICL/A195). A noninferiority analysis was also performed for the subgroup populations and noninferiority was not established for either subgroup (Table 3). However, latanoprost without BAK was less effective in lowering IOP in patients with high (29–35 mm Hg) baseline IOP compared with patients with low (22–28 mm Hg) baseline IOP. Time points where the 95% CI included 0, the 95% CI upper limit less than 1.5 mm Hg, and less than 1.0 mm Hg were 8/9, 12/0, and 6/0 for low IOP and high IOP, respectively (Table 3).

**TABLE 3.** Noninferiority Analysis Stratified by Intraocular Pressure at Baseline

Noninferiority Criteria	Low IOP Group (22–28 mm Hg) n=235 each^a^	High IOP Group (29–35 mm Hg) n=54 each^a^
N1: Time points where 95% CI includes 0	8/12	9/12
N2: Time points where 95% CI upper limit <1.5 mm Hg	12/12	0/12
N3: Time points where 95% CI upper limit <1.0 mm Hg	6/7	0/7

Data presented as (number of time points criterion met)/(minimum number of time points criterion must be met) and based on the ITT population.

For latanoprost without BAK to be considered noninferior to latanoprost with BAK, all three noninferiority criteria N1−N3 must be met for the minimum required time points.

N1, 95% CI includes 0 mm Hg; N2, the upper limit of the 95% CI is less than 1.5 mm Hg; N3, the upper limit of the 95% CI is less than 1 mm Hg.

a Each refers to each of the treatment groups, latanoprost BAK-free and reference.

BAK, benzalkonium chloride; CI, confidence interval; IOP, intraocular pressure; ITT, intent-to-treat.

### Safety

Distribution of treatment-emergent adverse events (TEAEs) was similar between treatment groups, and most TEAEs were mild in intensity. A total of 238 (82.4%) and 231 (79.9%) patients in the latanoprost without BAK and latanoprost with BAK groups reported an ocular TEAE, respectively (Table 4). More ocular TEAEs judged severe by the investigator were reported in the latanoprost without BAK versus the latanoprost with BAK group. The most common ocular TEAEs were eye pain, reported in 185 (64.0%) and 136 (47.1%) patients in the latanoprost without BAK and latanoprost with BAK groups, respectively; and ocular hyperemia, reported in 135 (46.7%) and 143 (49.5%) patients in the latanoprost without BAK and latanoprost with BAK groups, respectively. Systemic TEAEs were reported in 53 (18.3%) patients in the latanoprost without BAK group and 46 (15.9%) patients in the latanoprost with BAK group. Three patients were withdrawn from the study because of a TEAE, 1 in the latanoprost without BAK group and two in the latanoprost with BAK group.

**TABLE 4.** Summary of TEAEs and Most Commonly Occurring TEAEs

TEAEs	Latanoprost Without BAK (n=289)	Latanoprost With BAK (n=289)
Ocular		
Total	238 (82.4)	231 (79.9)
Treatment-related	230 (79.6)	222 (76.8)
Severe	18 (6.2)	8 (2.8)
Severe treatment-related	17 (5.9)	6 (2.1)
Leading to study medication discontinuation	1 (0.3)	2 (0.7)
Systemic		
Total	53 (18.3)	46 (15.9)
Treatment-related	7 (2.4)	9 (3.1)
Severe	3 (1.0)	5 (1.7)
Severe treatment-related	1 (0.3)	0
Serious	4 (1.4)	3 (1.0)

Most commonly occurring TEAEs	Latanoprost Without BAK (n=289)	Latanoprost With BAK (n=289)
Ocular, occurring in ≥5%		
Total	238 (82.4)	231 (79.9)
Eye pain	185 (64.0)	136 (47.1)
Ocular hyperemia	135 (46.7)	143 (49.5)
Conjunctival hyperemia	58 (20.1)	55 (19.0)
Eye discharge	39 (13.5)	41 (14.2)
Growth of eyelashes	27 (9.3)	36 (12.5)
Eye pruritus	16 (5.5)	14 (4.8)
Eyelash thickening	15 (5.2)	17 (5.9)
Systemic, occurring in ≥2%		
Any SOC	53 (18.3)	46 (15.9)
Infections and infestations	20 (6.9)	12 (4.2)
Skin and subcutaneous tissue disorders	10 (3.5)	5 (1.7)
Musculoskeletal and connective tissue disorders	7 (2.4)	2 (0.7)
Nervous system disorders	4 (1.4)	7 (2.4)
Vascular disorders	1 (0.3)	6 (2.1)

A patient with two or more TEAEs in a category was counted only once for that category. Medical Dictionary for Regulatory Activities version 12.0 was used. Data shown as n (%) and based on the safety population.

BAK, benzalkonium chloride; TEAE, treatment-emergent adverse event.

## DISCUSSION

The current study evaluated the noninferiority of latanoprost without BAK versus latanoprost with BAK in patients with OAG or OHT. Although not all criteria for noninferiority were met, patients receiving latanoprost without BAK achieved a substantial reduction of IOP from baseline, which was maintained for the 12-week duration of the study. Subgroup analysis of IOP change from baseline by baseline low (22–28 mm Hg) or high (29–35 mm Hg) IOP suggested latanoprost without BAK was less effective in lowering IOP in patients in the high IOP than in the low IOP baseline groups. However, the small sample size (n=54 in the high IOP and n=254 in the low IOP group) precluded the study from drawing meaningful conclusions. Future investigations to confirm this finding are warranted.

TEAEs were mostly mild with comparable numerical incidence between latanoprost without BAK (n=230 [79.6%]) and latanoprost with BAK (n=222 [76.8%]). No important safety concerns between treatment groups were found for ocular TEAEs, including eye pain, ocular and conjunctival hyperemia, visual acuity, eye lid and margin, cornea, anterior chamber, eye pruritus, visual field, and iris and eyelash changes. The latanoprost without BAK treatment arm had numerically more patients with eye pain, conjunctival hyperemia, and eye pruritis versus the latanoprost with BAK treatment group (n=185 vs n=136, n=58 vs n=55, and n=16 vs n=14, respectively). More ocular TEAEs judged severe by the investigator were identified in the latanoprost without BAK group (n=17 [5.9%]) compared with the latanoprost with BAK group (n=6 [2.1%]). Notably, in this study setting, the incidence reported for eye pain (64.0% and 47.1% for latanoprost without BAK and latanoprost with BAK, respectively) and for hyperemia (46.7% and 49.5% for latanoprost without BAK and latanoprost with BAK, respectively) were slightly higher compared with the ones observed in clinical trials conducted with latanoprost without BAK (55% and 41%).^8^ However, direct comparison across multiple trials is challenging given the inconsistency in the conditions and experimental designs adopted.

Patients with OAG or OHT tend to be older, who also are at higher risk for ocular surface disease. The longer a patient is on an IOP-lowering medication with BAK, the greater the risk of ocular surface disease, including dry eye disease.^10,11^ The BAK may be toxic to the corneal and conjunctival epithelium, and is also associated with a decrease in density of goblet cells, resulting in decreased mucin production and decreased tear film stability.^5,12^ A post-hoc analysis demonstrated a significant reduction in the ocular surface disease composite score (*P*<0.001) for patients receiving preservative-free latanoprost versus patients receiving preservative-containing latanoprost with no significant difference in change in IOP (*P*=0.312).^13^ Reducing exposure to BAK is important for maintaining the integrity of the ocular surface and tear film of patients who may require long-term topical therapy. Limitations of the current study include the 72-hr washout period for patients previously taking other prostaglandin analogs for the reduction of IOP. This short period raises the possibility that previous medication may interfere with baseline measurements in this study, and thus with the baseline reduction of IOP achieved by patients taking latanoprost without BAK. Another limitation of the study is the relatively short duration of 12 weeks. Many patients take IOP-reducing medications for years and it is possible that additional efficacy and/or safety advantages of a latanoprost without BAK formulation compared with formulation with BAK become more evident during long-term use. An open-label extension of the current study will examine the long-term safety of latanoprost without BAK for the reduction of IOP in patients with OAG and OHT.

A potential effect of BAK on latanoprost efficacy could also account for noninferiority criteria not being met. Instillation of BAK in the eye has been associated with increased rate of corneal drying.^12^ Moreover, superior penetration of ophthalmic formulations with BAK relative to formulations without BAK has been observed in multiple studies.^14–16^ It is hypothesized that BAK promotes drug penetration into the eye by loosening the tight junctions between corneal epithelial cells.^14–16^ If this was to be true, it would significantly affect the concentration of the drug reaching the aqueous humor, consequently increasing its efficacy.

Although noninferiority was not established, the substantial decrease in IOP, tolerability profile, and potential long-term risks associated with BAK exposure encourage the use of latanoprost 0.005% without BAK as a safe alternative to latanoprost 0.005% solution with BAK for the management of patients with OAG or OHT. However, further and larger studies are required to validate this finding.

## Supplementary Material

SUPPLEMENTARY MATERIAL
