# Supplementary material for: Noninferiority Study Comparing Latanoprost 0.005% Without Versus With Benzalkonium Chloride in Open-Angle Glaucoma or Ocular Hypertension
Source: Eye Contact Lens. 2021 Nov 17;48(4):149–54. doi: 10.1097/ICL.0000000000000860 (PMC8920005; doi:10.1097/ICL.0000000000000860)
Supplement: SUPPLEMENTARY MATERIAL [file ecl-48-149-s001.docx]

**Supplemental Table 1.** Noninferiority analysis comparing latanoprost without BAK vs latanoprost with BAK in the per-protocol population

|  |  |  | **Noninferiority criteria met** | | |
| --- | --- | --- | --- | --- | --- |
| **Time** | **Study day** | **IOP difference between treatment groups (95% CI)** | **N1** | **N2** | **N3** |
| 8 am | 7 | 0.31 (–0.26, 0.88) | Y | Y | Y |
|  | 28 | 0.65 (0.08, 1.22) | N | Y | N |
|  | 56 | 0.22 (–0.34, 0.78) | Y | Y | Y |
|  | 84 | 0.35 (–0.23, 0.93) | Y | Y | Y |
| 10 am | 7 | 0.40 (–0.15, 0.96) | Y | Y | Y |
|  | 28 | 0.43 (–0.15, 1.01) | Y | Y | N |
|  | 56 | 0.31 (–0.26, 0.88) | Y | Y | Y |
|  | 84 | 0.44 (–0.13, 1.01) | Y | Y | N |
| 4 pm | 7 | 0.39 (–0.16, 0.94) | Y | Y | Y |
|  | 28 | 0.82 (0.26, 1.39) | N | Y | N |
|  | 56 | 0.55 (–0.03, 1.13) | Y | Y | N |
|  | 84 | 0.71 (0.14, 1.28) | N | Y | N |
| **Number of times noninferiority criterion met:** | | | 9 | 12 | 6 |

For latanoprost without BAK to be considered noninferior to latanoprost with BAK, all 3 noninferiority criteria N1−N3 must be met for the minimum required time points.

N1, 95% CI includes 0 mmHg; N2, the upper limit of the 95% CI is <1.5 mmHg; N3, the upper limit of the 95% CI is <1 mmHg.

BAK, benzalkonium chloride; CI, confidence interval; IOP, intraocular pressure.
