# Supplementary figures and images for: Noninferiority Study Comparing Latanoprost 0.005% Without Versus With Benzalkonium Chloride in Open-Angle Glaucoma or Ocular Hypertension
Source: Eye Contact Lens. 2021 Nov 17;48(4):149–54. doi: 10.1097/ICL.0000000000000860 (PMC8920005; doi:10.1097/ICL.0000000000000860)

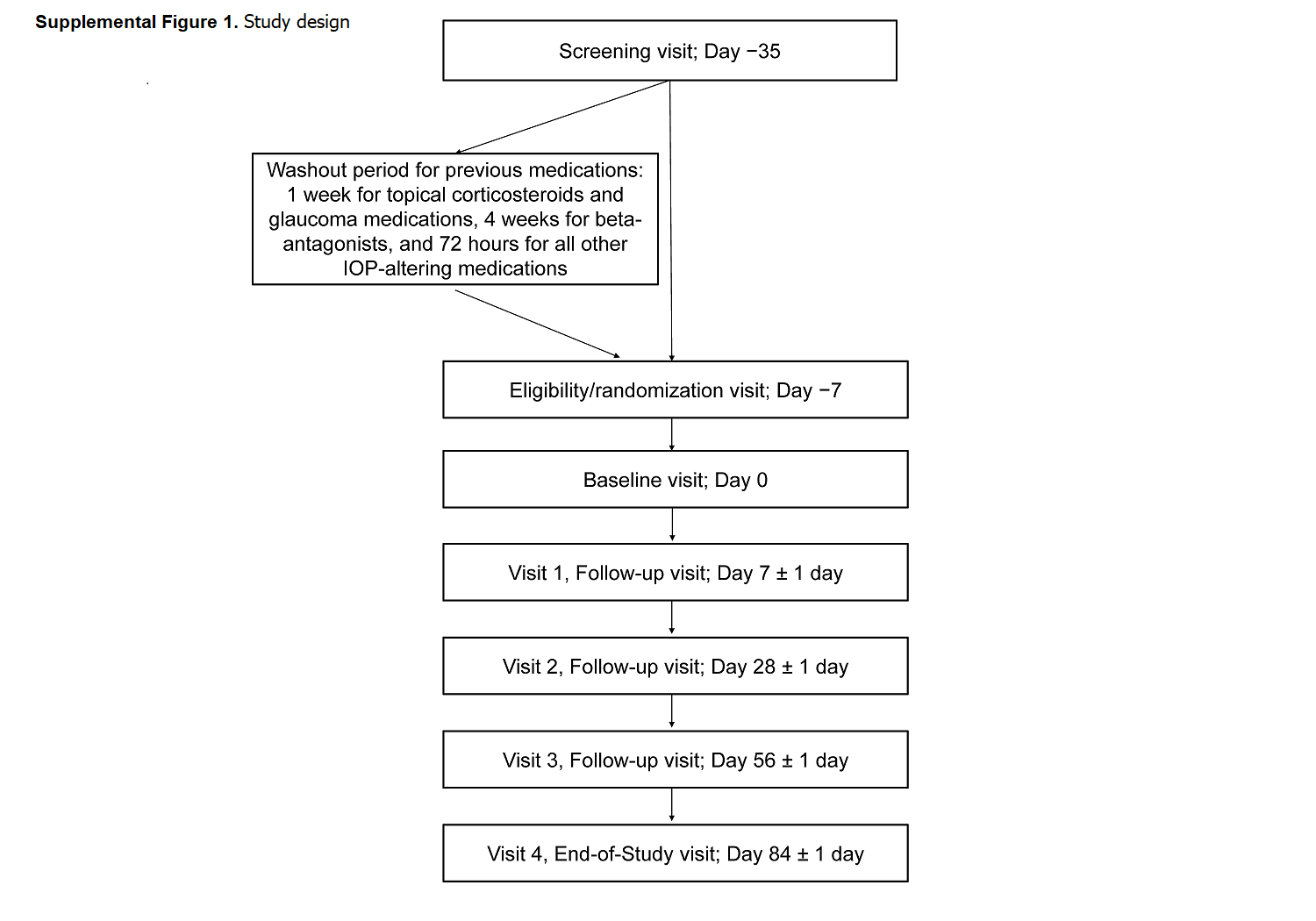

Supplement: SUPPLEMENTARY MATERIAL [file ecl-48-149-s002.tif]

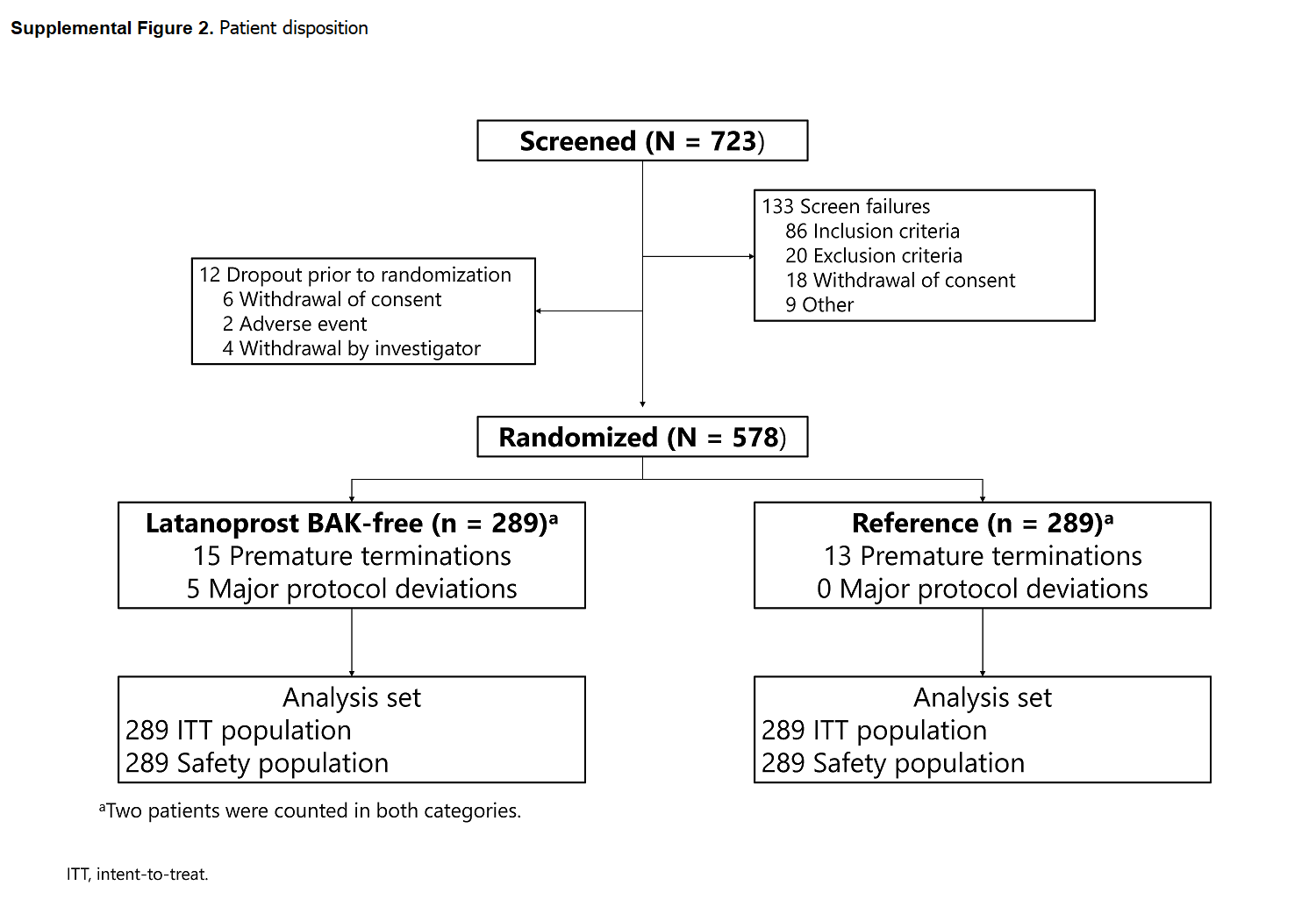

Supplement: SUPPLEMENTARY MATERIAL [file ecl-48-149-s003.tif]
